# Dexmedetomidine Inhibits Voltage-Gated Sodium Channels via *α*2-Adrenoceptors in Trigeminal Ganglion Neurons

**DOI:** 10.1155/2018/1782719

**Published:** 2018-09-02

**Authors:** Sang-Taek Im, Youn Yi Jo, Gayoung Han, Hyun Jung Jo, Yong Ho Kim, Chul-Kyu Park

**Affiliations:** ^1^Gachon Pain Center and Department of Physiology, College of Medicine, Gachon University, Incheon 21999, Republic of Korea; ^2^Department of Anesthesiology and Pain Medicine, Gachon University, Gil Medical Center, Incheon 21565, Republic of Korea; ^3^College of Art & Design, Kyung Hee University, Yongin 17104, Republic of Korea

## Abstract

Dexmedetomidine, an *α*2-adrenoceptor agonist, is widely used as a sedative and analgesic agent in a number of clinical applications. However, little is known about the mechanism by which it exerts its analgesic effects on the trigeminal system. Two types of voltage-gated sodium channels, Na_v_1.7 and Na_v_1.8, as well as *α*2-adrenoceptors are expressed in primary sensory neurons of the trigeminal ganglion (TG). Using whole-cell patch-clamp recordings, we investigated the effects of dexmedetomidine on voltage-gated sodium channel currents (*I*_Na_) via *α*2-adrenoceptors in dissociated, small-sized TG neurons. Dexmedetomidine caused a concentration-dependent inhibition of *I*_Na_ in small-sized TG neurons. *I*_Na_ inhibition by dexmedetomidine was blocked by yohimbine, a competitive *α*2-adrenoceptor antagonist. Dexmedetomidine-induced inhibition of *I*_Na_ was mediated by G protein-coupled receptors (GPCRs) as this effect was blocked by intracellular perfusion with the G protein inhibitor GDP*β*-S. Our results suggest that the *I*_Na_ inhibition in small-sized TG neurons, mediated by the activation of Gi/o protein-coupled *α*2-adrenoceptors, might contribute to the analgesic effects of dexmedetomidine in the trigeminal system. Therefore, these new findings highlight a potential novel target for analgesic drugs in the orofacial region.

## 1. Introduction

Dexmedetomidine is a potent and highly selective agonist of the *α*2-adrenoceptor with a wide range of effects that include sedation, anesthetic-sparing activity, analgesia, and adjuvant antinociception [1, 2]. *α*2-Adrenoceptors are widely distributed throughout the peripheral and central nervous system including primary afferents, spinal dorsal horns, and the brain stem, and their activation produces a variety of effects [3–6]. Three *α*2-adrenoceptor subtypes (*α*2A, *α*2B, and *α*2C) have been cloned, all of which are coupled to inhibitory G proteins and play an important role in the control of pain [7, 8]. Systemically administered dexmedetomidine increases the threshold of mechanical and thermal pain and produces antinociceptive effects, in humans and animals, suggesting that the *α*2-adrenoceptor may be involved in antinociception at the peripheral level [9–11]. Despite substantial evidence supporting an antinociceptive effect of dexmedetomidine in the dorsal root ganglion (DRG), the underlying mechanisms remain poorly understood in the orofacial region. [12–14].

The trigeminal ganglion (TG) is the counterpart of the DRG at the spinal level, and both areas have comparable neuronal populations. TG neurons are involved in pain sensation in the orofacial area. Neurons in TGs are also known to have nociceptors and neurochemical properties similar to that of DRG neurons [15]. Voltage-gated sodium channels (VGSCs) play an important role in action potential initiation and propagation in excitable cells, including sensory neurons in the TG and DRG, because they are responsible for the initial depolarization of the membrane. VGSCs in primary nociceptive neurons of the TG are involved in pain transduction and transmission processes in orofacial regions [16–18]. Therefore, controlling the excitability of nociceptive TG neurons by modulating VGSCs would provide a useful tool for the management of physiological or pathological pain in the orofacial area.

Recent studies have revealed that dexmedetomidine inhibits both tetrodotoxin-resistant and tetrodotoxin-sensitive sodium channels in DRG neurons [8, 19, 20]. However, it remains unclear whether dexmedetomidine can inhibit the function of VGSCs in neurons of the trigeminal system. In the present study, we investigated whether the peripheral dexmedetomidine-induced analgesia in the orofacial area might, in part, arise from suppression of VGSC activation via binding to Gi/o protein-coupled *α*2-adrenoceptors in small-sized TG neurons.

## 2. Materials and Methods

### 2.1. Animals

All surgical and experimental procedures were reviewed and approved by the Institutional Animal Care and Use Committee of the College of Medicine at Gachon University. C57BL/6 mice (male, 6–8 weeks) were purchased from OrientBio (Sungnam, Korea). Thirty mice were habituated for at least 1 week prior to experiments in a conventional facility with a 12 : 12 h light-dark cycle (lights on at 8:00 am) and had ad libitum access to food and water.

### 2.2. Preparation of Trigeminal Ganglion (TG) Neurons

TG neurons from C57BL/6 mice were prepared as previously described [18]. Briefly, TG kept at 4°C in Hank's Balanced Salt Solution (HBSS; Welgene, Daegu, Korea) were incubated in 2 mL HBSS containing 0.25% trypsin (Invitrogen, Carlsbad, CA, USA) at 37°C for 60 min. Cells were washed, triturated with a flame-polished Pasteur pipette, and placed on glass coverslips coated with 0.5 mg/mL poly-L-ornithine (Sigma-Aldrich, St. Louis, MO, USA). The cells were maintained at 37°C in a 5% CO_2_ incubator and were used for recordings within 8 h after being plated.

### 2.3. Whole-Tissue Reverse Transcription Polymerase Chain Reaction (RT-PCR)

Total RNA was extracted from murine TGs using the alphaPrep Total RNA mini kit (Alph*α*gene, Sungnam, Korea) according to the manufacturer's instructions. The RNA was subjected to RT-PCR using oligo (dT) reverse transcriptase primers and SuperScript III reverse transcriptase (Invitrogen, Carlsbad, CA, USA) and was kept at 37°C for an hour for the reverse transcription reaction. Subsequently, PCR amplifications were performed with primers shown in Table 1. The PCR products were then run on an ethidium bromide-stained 1.2% agarose gel.

### 2.4. Whole-Cell Patch-Clamp Recordings

Whole-cell voltage- and current-clamp recordings were performed at 24–28°C to measure currents and action potentials, respectively, using an Axopatch 200B amplifier (Axon Instruments, Union City, CA, USA). The patch pipettes were pulled from borosilicate capillaries (Chase Scientific Glass Inc., Rockwood, CA, USA). When filled with the pipette solution, the resistance of the pipettes was 4-5 MΩ. The recording chamber (volume 300 *μ*L) was continuously superfused (2-3 mL/min). Series resistance was compensated for (>80%), and leak subtraction was performed. Data were low-pass filtered at 2 kHz and sampled at 10 kHz. pClamp8 (Axon Instruments) software was used for experiments and analysis. The pipette solution for voltage-clamp experiments contained (in mM) 135 CsCl, 30 CsOH, 2 Mg-ATP, 5 EGTA, and 10 HEPES, adjusted to pH 7.4 with CsOH, with an osmolarity of 295–300 mOsm. In some cases, guanosine 5′-[*β*-thio] diphosphate trilithium salt (GDP*β*-S, 2.5 mM) was included in the intracellular solution to block G protein-coupled receptors (GPCRs). The extracellular solution for voltage-clamp experiments contained (in mM) 140 NaCl, 5 KCl, 1 MgCl_2_, 10 HEPES, 10 glucose, and 2 EGTA, adjusted to pH 7.4 with NaOH, with an osmolarity of 300–310 mOsm. Voltage-clamp experiments were performed at a holding potential of −60 mV. The pipette solution for current-clamp experiments contained (in mM) 145 K-gluconate, 2 MgCl_2_, 1 CaCl_2_, 10 EGTA, 5 HEPES, and 5 K_2_ATP, adjusted to pH 7.3-7.4 with KOH, with an osmolarity of 300 mOsm. The extracellular solution for current-clamp experiments contained (in mM) 140 NaCl, 5 KCl, 2 CaCl_2_, 1 MgCl_2_, 10 HEPES, and 10 glucose, adjusted to pH 7.4 with NaOH, with an osmolarity of 300–310 mOsm. IC_50_ values were calculated by normalizing peak current amplitudes at different drug concentrations to the value obtained for the control solution. Furthermore, the data were fitted to the Hill equation [12].

### 2.5. Drugs

All chemicals were purchased from Sigma-Aldrich (St. Louis, MO, USA). Dexmedetomidine, yohimbine hydrochloride, and GDP*β*-S were dissolved in distilled water to make a stock solution. The drugs were diluted to their final concentrations in the extracellular solution and then administered by gravity through a bath perfusion system.

### 2.6. Statistical Analysis

All data are expressed as the mean ± standard error of the mean (SEM). One-way analysis of variance (ANOVA) or unpaired Student's *t*-test was used to determine statistical difference using Origin 6.0 (Microcal Software Inc., Northampton, MA, USA). Differences were considered to be significant with *p* < 0.05.

## 3. Results

### 3.1. Gene Expression of Voltage-Gated Sodium Channels and the *α*2-Adrenoceptor in the Trigeminal Ganglia

**Figure 1:**
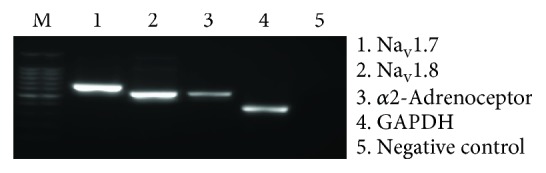
Expression of Na_v_1.7, Na_v_1.8, and the *α*2-adrenoceptor in TG neurons.

Two types of VGSCs, Na_v_1.7 and Na_v_1.8, are known to contribute to pain transduction in nociceptive neurons [21, 22]. Therefore, we investigated whether TG neurons expressed Na_v_1.7, Na_v_1.8, and *α*2-adrenoceptor mRNAs using RT-PCR. Size separation of PCR products from murine TG by electrophoresis showed the presence of 649, 544, and 538 bp amplicons, as expected for Na_v_1.7, Na_v_1.8, and *α*2-adrenoceptor subtype A, respectively (Figure 1).

### 3.2. Dexmedetomidine Inhibits Voltage-Gated Sodium Channel Currents (*I*_Na_) and Action Potentials (APs) in Small-Sized TG Neurons

**Figure 2:**
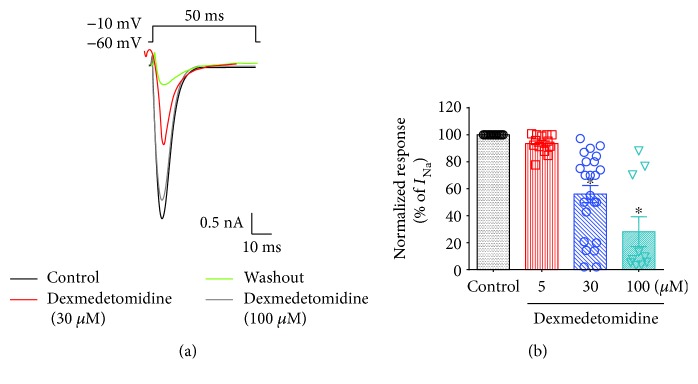
Effects of dexmedetomidine on voltage-gated sodium channel currents (*I*_Na_) in small-sized TG neurons. (a) Dexmedetomidine suppresses *I*_Na_ in a concentration-dependent manner. Traces showing *I*_Na_, the effects of dexmedetomidine (30, 100 *μ*M), and the washout. (b) Percent inhibition of *I*_Na_ by dexmedetomidine (5 *μ*M, *n* = 15/25; 30 *μ*M, *n* = 20/32; 100 *μ*M, *n* = 10/16). Results are presented as mean ± SEM. ^∗^*p* < 0.05; *t*-test versus control; *n* = 10–20 neurons per group.

**Figure 3:**
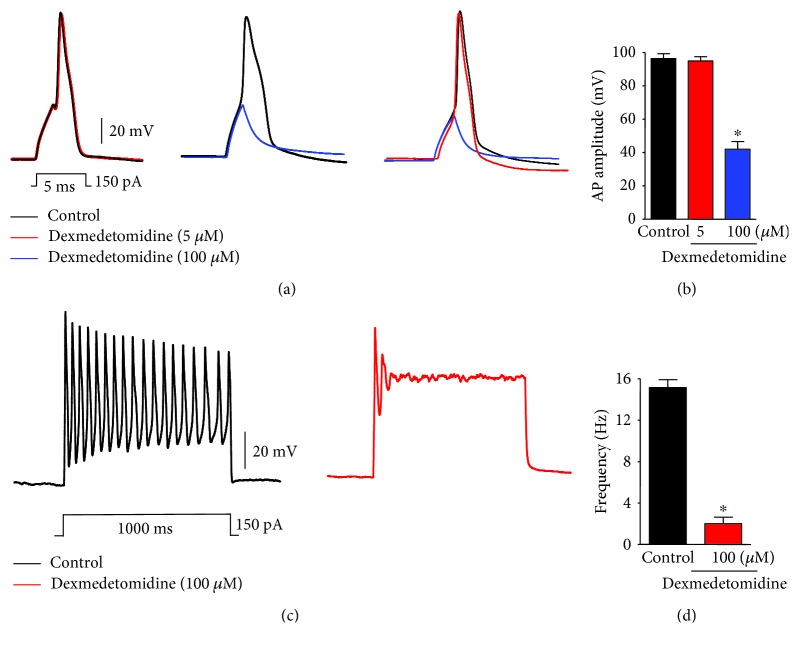
Effects of dexmedetomidine on action potentials (APs) in small-sized TG neurons. (a) Dexmedetomidine (100 *μ*M) suppresses APs following current injection (5 ms, 150 pA) in small-sized TG neurons. Traces of single action potentials before (control) and after treatment with dexmedetomidine (5 and 100 *μ*M). (b) Percent inhibition of AP amplitude by dexmedetomidine (5 and 100 *μ*M). ^∗^*p* < 0.05; *t*-test versus control; *n* = 15 and 20 neurons per group, respectively. n.s.: no significance. (c) Dexmedetomidine inhibits the AP frequency after current injection (1 s, 150 pA) in small-sized TG neurons. Traces of action potentials before (control) and after treatment with dexmedetomidine (100 *μ*M). (d) Dexmedetomidine (100 *μ*M) markedly suppresses 90% of the AP frequency after current injection (150 pA) ^∗^*p* < 0.05; *t*-test versus control; *n* = 15 and 20 neurons per group, respectively

VGSCs are mainly expressed in small-sized nociceptive sensory neurons and play an important role in regulating APs [21, 22]. Because both Na_v_1.7 and Na_v_1.8 are responsible for the initial depolarization phase involved in the generation of APs [23], we examined whether dexmedetomidine could modulate *I*_Na_ and APs in small-sized TG neurons. To test this, we recorded *I*_Na_ and APs in these small-sized TG neurons (10 to 25 *μ*m diameter) using whole-cell voltage- and current-clamp electrophysiology, respectively. *I*_Na_ inhibition by dexmedetomidine was concentration dependent (IC_50_ = 33 *μ*M; Figures 2(a) and 2(b)). Dexmedetomidine, at a concentration of 100 *μ*M, significantly inhibited *I*_Na_ (72 ± 3%, *n* = 10/16) (Figures 2(a) and 2(b)). Dexmedetomidine also inhibited the generation of APs following current injection in a concentration-dependent manner (Figures 3(a) and 3(b)). As expected, dexmedetomidine markedly suppressed the AP frequency after current injection (Figures 3(c) and 3(d)).

### 3.3. Dexmedetomidine Inhibits *I*_Na_ via *α*2-Adrenoceptors in Small-Sized TG Neurons

**Figure 4:**
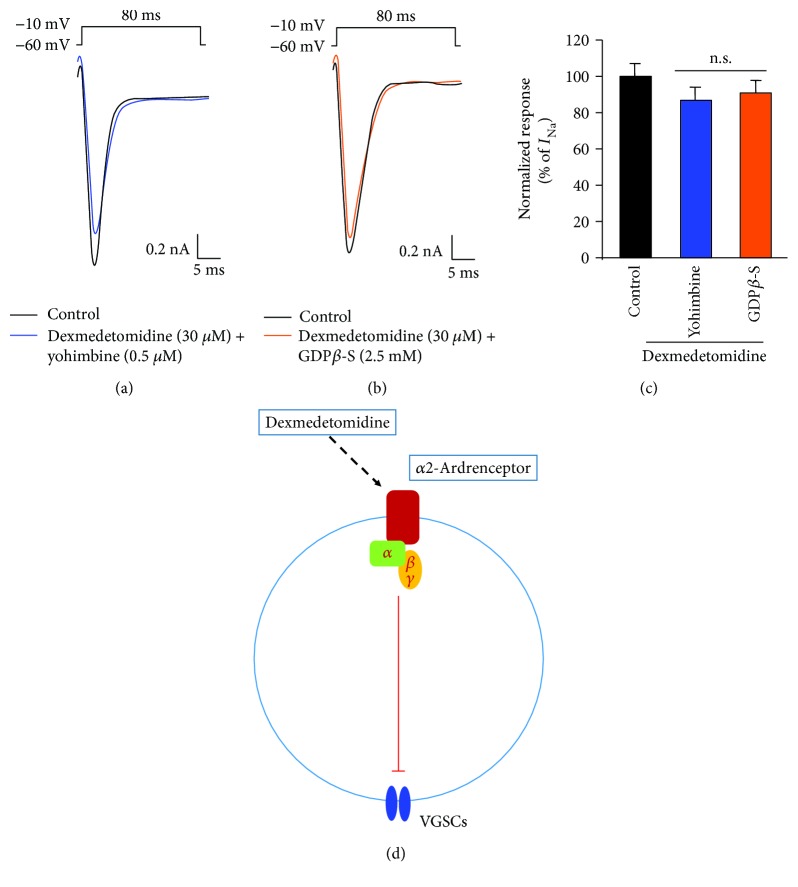
Effects of dexmedetomidine on *I*_Na_ via Gi/o protein-coupled *α*2-adrenoceptors in small-sized TG neurons. (a) Treatment with yohimbine (0.5 *μ*M, 2 min) suppresses the inhibitory effects of dexmedetomidine on *I*_Na_ in small-sized TG neurons (*n* = 15). (b) Intracellular perfusion with GDP*β*-S (2.5 mM, 8 min) blocks inhibition of *I*_Na_ by dexmedetomidine (*n* = 10). (c) Summary of inhibitory yohimbine and GDP*β*-S effects on the dexmedetomidine-mediated inhibition of *I*_Na_. Results are presented as mean ± SEM. n.s.: no significance. (d) Working hypothesis for the inhibition of *I*_Na_ by dexmedetomidine via Gi/o protein-coupled *α*2-adrenoceptors in small-sized TG neurons. Sensory TG neurons do not only express neuronal VGSCs and Gi/o protein-coupled *α*2-adrenoceptors. Stimulation of the *α*2-adrenoceptor by dexmedetomidine suppresses the activity of VGSCs by inhibitory GPCR pathways in dissociated small-sized TG neurons.

Next, we investigated whether *I*_Na_ inhibition by dexmedetomidine was dependent on the Gi/o protein-coupled receptor (GPCR) signaling pathway mediating *α*2-adrenoceptor activation. When either the *α*2-adrenoceptor inhibitor, yohimbine (0.5 *μ*M, 2 min), or the G protein inhibitor, GDP*β*-S (2.5 mM, 8 min), was used, the inhibitory effect of dexmedetomidine on *I*_Na_ was abolished (Figures 4(a)–4(c)). This indicates that the activation of a GPCR signaling pathway by *α*2-adrenoceptors is integrally involved in the dexmedetomidine-induced inhibition of *I*_Na_ (Figure 4(d)).

## 4. Discussion

In this study, we demonstrate that the neuronal VGSCs, Na_v_1.7 and Na_v_1.8, which are the primary pain-sensing elements in pain sensation, and the *α*2-adrenoceptor, which is a selective receptor of dexmedetomidine, are expressed at the mRNA level in sensory TG neurons. We also show that dexmedetomidine inhibits *I*_Na_ and APs, in a concentration-dependent manner, through activation of *α*2-adrenoceptors expressed in small-sized TG neurons. Our results suggest that this inhibition occurs through an intracellular signaling mechanism activated by Gi/o protein-coupled *α*2-adrenoceptors, and that, through the inhibitory effect on *I*_Na_ in primary sensory neurons in the trigeminal system, dexmedetomidine can effectively inhibit orofacial pain. These new findings highlight a potential novel drug target for analgesia in the orofacial region.

Dexmedetomidine, a potent and highly selective agonist of the *α*2-adrenoceptor, has been widely used for its sedative and analgesic effects [24]. *α*2-Adrenoceptors, which are activated by dexmedetomidine, are most commonly found in brainstem nuclei, neurons in the superficial laminae of the spinal cord, and the peripheral nerve terminal [3–5]. Moreover, it was reported that the *α*2-adrenoceptor was expressed by more than 60% of neurons in the TG and over 80% of neurons in the DRG [5, 6]. Recent studies demonstrate the antinociceptive mechanism of dexmedetomidine in the somatosensory system, specifically at the spinal cord and peripheral nervous system [8, 19, 20, 25]. Several studies specifically targeted ion channels (VGSCs, hyperpolarization-activated cyclic nucleotide-gated channels) in the dorsal root ganglion that are primarily related to nociception. These studies show that the mechanism of action of dexmedetomidine is related to the suppression of these channels [8, 19, 20, 26]. This suggests that dexmedetomidine blocks pain in the somatic system at the level of the peripheral nervous system. Moreover, dexmedetomidine has been shown to affect another VGSC, Na_V_1.5, in cardiac cells [27]. However, there is a lack of research on the antinociceptive effects of dexmedetomidine in the orofacial region, which involve the trigeminal system.

Nociception within the trigeminal system could be different from other, common, found pain-processing mechanisms. Although there are anatomical and functional similarities between the spinal and trigeminal somatosensory systems, the segmental distribution of the somatic sensory input is relatively less organized in the trigeminal sensory system. In addition, the distance between the ganglion and its target in the trigeminal system is much shorter than that in other parts of the somatosensory system [28, 29]. There are several different functional types of TG neurons in the trigeminal sensory system, which is reflected in the heterogeneity of cell bodies in this area. TG neurons can vary greatly, both in cell body size and in the expression of ion channels and other proteins [18, 30]. Nociceptive TG neurons are unmyelinated C-fibers, small in size that express VGSCs [16]. VGSCs, such as Na_v_1.7 and Na_v_1.8, are the main ion channels involved in the generation and propagation of APs [21–23]. Generation and propagation of APs are required for pain sensation in the trigeminal system [17, 18]. By testing whether dexmedetomidine inhibits VGSCs within the trigeminal system, we investigated its potential as a new medical treatment for orofacial pain.

We first confirmed whether Na_v_1.7 and Na_v_1.8, which are the major neuronal VGSCs in TG neurons, and the *α*2-adrenoceptor, which is a receptor of dexmedetomidine, could be detected by RT-PCR. Our results showed that Na_v_1.7, Na_v_1.8, and the *α*2-adrenoceptor are all expressed in the TG (Figure 1). This is consistent with previous research on the somatic system that suggests an inhibitory mechanism of dexmedetomidine on *I*_Na_ through the activation of *α*2-adrenoceptors in the trigeminal system.

There are two general classes of sodium currents in small-sized TG neurons: one is blocked by TTX (TTX-sensitive or TTX-s *I*_Na_), and the other is insensitive to TTX (TTX-resistant or TTX-r *I*_Na_) [17]. In small-sized TG neurons, TTX-s *I*_Na_ and TTX-r *I*_Na_ are generated by activation of Na_v_1.7 and Na_v_1.8, respectively. Na_v_1.7 and Na_v_1.8 are expressed together in the trigeminal system, and, by simultaneous activation, they generate APs that are critical for pain sensation [17, 23]. Using whole-cell patch-clamp recording experiments, we confirmed that dexmedetomidine inhibits *I*_Na_ in small-sized TG neurons in a concentration-dependent manner (Figures 2(a) and 2(b)). Specifically, a concentration of 100 *μ*M dexmedetomidine significantly decreased *I*_Na_ amplitudes in small-sized TG neurons (Figures 2(a) and 2(b)). This observation confirms that both the TTX-s and TTX-r *I*_Na_ are suppressed by dexmedetomidine. Thus, the inhibitory effect of dexmedetomidine on both TTX-s and TTX-r *I*_Na_ may contribute to its analgesic activity. Dexmedetomidine also prevents the generation of APs following current injection in small-sized TG neurons in a concentration-dependent manner, again via *I*_Na_ inhibition (Figures 3(a) and 3(b)). As expected, dexmedetomidine markedly decreases the AP frequency after current injection (Figures 3(c) and 3(d)).

Our results suggest that by activating *α*2-adrenoceptors expressed in small-sized TG neurons, dexmedetomidine can inhibit orofacial pain through intracellular mechanisms that inhibit neuronal VGSCs and APs within the trigeminal sensory system. The mechanism by which dexmedetomidine-stimulated *α*2-adrenoceptors inhibit *I*_Na_ in the trigeminal system has not been well described yet. Since *α*2-adrenoceptors activate intracellular signaling through specific GPCR pathways [7], we tested the effect of yohimbine, an *α*2-adrenoceptor inhibitor, and GDP*β*-S, a G protein inhibitor, on dexmedetomidine-induced inhibition of *I*_Na_ in small-sized TG neurons. Both yohimbine and GDP*β*-S completely blocked the *I*_Na_ inhibition by dexmedetomidine (Figures 4(a)–4(c)). Our results demonstrate that the inhibitory effects of dexmedetomidine on *I*_Na_ in this neuronal population are likely to be mediated by the activation of specific Gi/o-coupled receptors (Figure 4(d)). Therefore, dexmedetomidine not only acts as an endogenous activator of *α*2-adrenoceptors but may also serve as an endogenous selective inhibitor of VGSCs in the trigeminal system.

In summary, dexmedetomidine is a sedative agent with selective antinociceptive effects. The current study demonstrates the inhibition of *I*_Na_ by dexmedetomidine in primary sensory TG neurons and suggests that activation of Gi/o-coupled *α*2-adrenoceptors might be the mechanism underlying the analgesic activity of this compound. Inhibition of peripheral *I*_Na_ indicates that the analgesic effect of dexmedetomidine might be independent of its sedative effect, which is exerted on the central nervous part of the trigeminal system. This independent mechanism suggests that dexmedetomidine can be a potential local analgesic agent for the treatment of *I*_Na_-mediated pain in the trigeminal system, including orofacial hypersensitivity.

## Figures and Tables

**Table 1:** List of DNA primer sequences designed for RT-PCR.

Target gene (product lengths)	Outer forward	Outer reverse	GenBank number
Na_v_1.7 (649 bp)	GCTGATCTCTCTCAGGCATTAC	CATCTCAAAGTCGTCCTCACTC	NM_018852.2
Na_v_1.8 (544 bp)	CCTCATCTTCTGGCTCATCTTC	CACGAAGCCCTGGTACTTATT	AY538273.1
*α*2-Adrenoceptor (538 bp)	CTCGCTGAACCCTGTTATCTAC	GACCGCCCTGAATGATCTTTAT	NM_007417.4
GAPDH	AGCCTCGTCCCGTAGACAAAA	TTTTGGCTCCACCCCTTCA	XM_001473623

## Data Availability

The data used to support the findings of this study are included within the article.
